# Short Placental Telomere was Associated with Cadmium Pollution in an Electronic Waste Recycling Town in China

**DOI:** 10.1371/journal.pone.0060815

**Published:** 2013-04-02

**Authors:** Shuiqin Lin, Xia Huo, Qingying Zhang, Xiaojuan Fan, Li Du, Xijin Xu, Shaoshan Qiu, Yuling Zhang, Yun Wang, Jiang Gu

**Affiliations:** 1 Guangdong Provincial Key Laboratory of Infectious Diseases and Molecular Immunopathology, Shantou University Medical College, Shantou, China; 2 Department of Pathology, School of Basic Medical Science, Peking University, Beijing, China; 3 Analytical Cytology Laboratory, Shantou University Medical College, Shantou, China; 4 Department of Preventive Medicine of Shantou University Medical College, Shantou, China; Tulane University Health Sciences Center, United States of America

## Abstract

In Guiyu, an electronic waste recycling site near Shantou, Guangdong province, China, primitive ways of e-waste processing have caused severe cadmium and lead pollution to the local residents. However, the possible effects of cadmium or lead pollution to genomic integrity of the local residents have not been investigated. We examined the possible relationship between cadmium and lead concentrations in placenta and placental telomere length in Guiyu and compared the data with that of a non-polluted town. Graphite furnace atomic absorption spectrometry and real-time PCR were used to determine placental cadmium and lead concentrations, and placental telomere length. We found that placental cadmium concentration was negatively correlated with placental telomere length (r = −0.138, p = 0.013). We also found that placental cadmium concentration of 0.0294 µg/g might be a critical point at which attrition of placental telomere commenced. No significant correlation between placental lead concentration and placental telomere length was detected (r = 0.027, p = 0.639). Our data suggest that exposure to cadmium pollution during pregnancy may be a risk factor for shortened placental telomere length that is known to be related to cancer development and aging. Furthermore, grave consequence on the offspring from pregnancies in e-waste polluted area is indicated.

## Introduction

**Figure 1:**
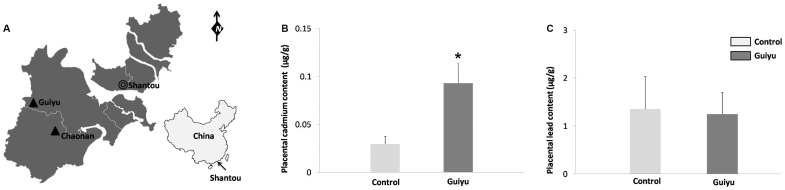
Map of the sampling sites, placental cadmium and lead concentration in Guiyu and the control group. (A) Map of the sampling sites. (B) Placental cadmium concentration in Guiyu and the control group. *: p<0.01, Guiyu group vs. the control group. (C) Placental lead concentration in Guiyu and the control group. Data are presented as median±SEM.

Guiyu, a town with a population of 132,000 and about 40 KM west of Shantou city, Guangdong province, China (Figure 1A), is one of the biggest recycling centers for e-waste in the world with a 30-year history of operation. About 60–80% of the local families have engaged in the process of e-waste recycling operated in family-run workshops often without the most basic safety precaution or any environmental protection measure. The techniques and facilities used in the process of e-waste recycling were primitive and uncontrolled [1]. It has been reported that significantly high concentration of toxic heavy metals and organic pollutants were found in hair, peripheral blood, milk, placenta, umbilical cord blood and meconium of Guiyu residents [1], [2]. A study by Xu et al reported that stillbirth rate, LBW (Low Birth Weight) rate and IUGR (Intrauterine Growth Retardation) rate in Guiyu were all significantly higher than that of Xiamen, a city 300 KM north of Guiyu and free of e-waste recycling [3]. Close relationships between children temperament alterations and e-waste recycling activities in Guiyu were also noted [4]. Cadmium and lead were widely used as important raw materials in the manufacture of various electronic devices. Cadmium is known to accumulate in human body, causing damages to kidneys and bone structures [5]–[7]. Lead can also build up in human body and has irreversible toxic effects on human nervous system, especially on the developing nervous system of children [8].

Placenta functions as a barrier and a bridge simultaneously between fetal and maternal circulation and is critically important for fetal development [9]. Placental insufficiency or malfunction has been linked to adverse birth outcomes [9], [10]. Our previous studies found that cadmium and lead were the predominant toxic heavy metals in the placentas of Guiyu town [11]. Cadmium has been shown to structurally and functionally affect the development of placenta, yolk sac and embryo, and even cause embryonic absorption, malformation and death [12], [13]. Intrauterine lead exposure may result in spontaneous abortion or fetal death in extreme cases [14]–[17]. Placenta has been used by many researchers as an indicator organ to study the mechanism of metal induced toxicity [18], [19].

Telomere is terminal structure of eukaryotic chromosomes, comprising of hundreds of thousands of highly repetitive tandem repeats (TTAGGG in human) and associated proteins. Telomere serves as a cap at the end of chromosomes to maintain the integrity of chromosomes and the stability of genome, and to prevent end-to-end chromosomal fusions [20]. Telomere is of great importance in placental development and maturation. Shortened placental telomere is thought to cause adverse birth outcomes, e.g. fetal growth restriction and low birth weight [21], [22]. Shortened telomere is also related to increased cancer risk and aging [23]–[26].

However, the relation between tissue cadmium and lead concentrations and telomere has not been investigated in human. In this study, we examined the relationships between placental cadmium and lead concentrations and telomere length in term placentas collected from deliveries by Guiyu residents and from other areas without known e-waste pollution.

## Materials and Methods

### Recruitment of study subjects

Healthy puerperae, were recruited from Guiyu and two other e-waste free towns, Chaonan (20 km from Guiyu) and Shantou urban district (40 km from Guiyu) where no e-waste recycling workshops were present (Figure 1A). Two hundred twenty seven (227) puerperae from the local hospital of Guiyu were recruited for the period of 2006 to 2010. Concurrently 93 puerperae from Chaonan Minsheng hospital and the local hospitals of Shantou urban district were recruited as the control group. The traffic density, cultural background, lifestyle, and socioeconomic status of the three sites were similar. Written informed consents were obtained from participants in accordance with the Declaration of Helsinki. This study was approved by the Human Ethic Committee of Shantou University Medical College.

### Questionnaire survey

A questionnaire designed to unveil the exposure of puerpera to cadmium and lead was completed by each participant. The questionnaire included three parts. The first part was about the general condition of neonate's parents, focusing on their living and working environment. The main aim of this part was to assess the association of environmental cadmium and lead exposure with placental telomere length. The second part involved the physical conditions of the puerpera and other confounding factors that might affect placental telomere length, e.g. drinking and smoking. The third part concerned the general health of the neonate.

### Collection of placental samples

Immediately after delivery, a piece of 2 cm^3^ central region placenta (cotyledon) was dissected with titanium tools, and the decidua was then removed. The placental sample was rinsed to remove maternal blood, and then stored in polyethylene tube to prevent metal contaminations. Each tube was properly labeled with corresponding identification code and preserved on ice in a portable refrigerator. Samples were transported to laboratory as quickly as possible and kept at −20°C before assay.

### Measurement of placental cadmium and lead concentrations

To determine placental cadmium concentration, a tiny piece (∼2×2×2 mm^3^) of placental tissue was first cut and weighted, followed by digestion in nitric acid at 80°C water-bath for 1 hour. H_2_O_2_ was added next and the final volume was raised to 3 ml with deionized water. To determine placental lead concentration, a separate piece of placental sample was dissected and processed similarly except that the sample piece was first dried overnight before weighting and digestion. Cadmium and lead concentrations were then determined as described by Guo et al [11]. They were measured in the Analytical Laboratory of Shantou University Medical College with GFAAS, which consisted of an autosampler (MPE60), with an injection volume set at 10 µL. The main parameters used for Pb determination were a wavelength of 283.3 nm, a lamp current of 4.0 mA, a slit width of 0.8 nm, drying at 90°C, 100°C, and 120°C, ashing at 950°C, and atomization at 1500°C. The parameters for Cd analysis were a wavelength of 228.8 nm, a current of 4.0 mA, a slit width of 0.8 nm, drying at 90°C and 105°C, ashing at 900°C, and atomization at 1300°C.

### Genomic DNA extraction from placenta

For each placenta, a piece of tissue about 50 mg was quickly dissected with one set of tools to prevent cross contamination, and pulverized thoroughly in a glass homogenizer. Placental genomic DNA was then extracted with a commercial kit (Universal genomic DNA extraction kit ver. 3.0, Takara, Dalian, China) according to manufacturer's instruction. Concentration of placental genomic DNA was determined using a Nanodrop 2000c spectrophotometer (Thermo Scientific, Delaware, USA), subsequently aliquoted and preserved in a −20°C freezer until assay. To assure that digestion of intact DNA of placenta did not lead to preferential loss of the telomere and a skewed data distribution of telomere length, One-Sample Kolmogorov-Smirnov Test was used to test the normality of data distribution of telomere length as described in the Data analysis section.

### Assessment of placental telomere length

Placental telomere length was determined with a real-time PCR-based telomere assay previously described by Richard M. Cawthon [27] with minor modifications. The gene 36B4 was selected as the single copy gene. The reference DNA was composed of ten randomly selected DNA samples in this study.

Briefly, for each DNA sample, determination of relative telomere repeat copy number and relative copy number of single copy gene were conducted in the following two 20 µl PCR reaction systems, one with telomere primer pair (Tel 1 and Tel 2) and another with 36B4 primer pair (36B4u and 36B4d ). PCR reaction system was constructed using the SYBR Premix Ex Taq™ (Tli RNaseH Plus) kit (Takara, Dalian, China). The 20 µl PCR reaction system with telomere primer pair contained a final concentration of 300 nM Tel 1 primer, 900 nM Tel 2 primer, 1.5 ng/μl genomic DNA template, 1X SYBR premix Ex Taq™ mixture and 1X ROX reference Dye. The 20 µl PCR reaction system with 36B4 primer pair contained a final concentration of 300 nM 36B4u primer, 500 nM 36B4d primer, 1.5 ng/μl genomic DNA template, 1X SYBR premix ex Taq™ mixture and 1X ROX reference dye.

All the DNA samples were assessed in triplicate. Sequence of primers and their thermal cycling profiles were listed in Table S1. In each run for telomere and 36B4 amplification, a standard curve and three negative controls were included. To produce the standard curve, one reference DNA was serially diluted with deionized water by 2-fold per dilution to create five concentrations of DNA ranging from 0.17 to 2.75 ng/μl.

The mean R^2^ for all standard curves was >0.98. For each DNA sample, the relative telomere repeat copy number and the relative copy number of single copy gene were respectively calculated against the corresponding standard curve. Telomere length was expressed as the ratio of these two values (T/S ratio), which reflected the average telomere repeat copy number of each DNA sample calculated relative to the reference DNA. The variation of T/S ratio of three assessments was less than 10% for all the placental DNA samples tested, and the inter-run and intra-run variations of this assay were 6.495% and 7.818% respectively.

### Measurement of terminal restriction fragment length

Terminal restriction fragment (TRF) length assay based on southern blotting was performed as previously described [28]. Genomic DNA (1 µg) was first digested with Hinf I and RsaI, and then parallelly resolved with electrophoresis in 0.8% agarose gel with digoxigenin-labeled λ/Hind III digested DNA marker. Gel was subjected to denaturation and neutralization and the genomic DNA was transferred to PVDF membrane. Hybridization was performed by incubating the membrane in hybridization buffer containing 10 pmol/ml digoxigenin-labeled telomeric oligonucleotide (TTAGGG) at 42°C for 3 h. After washing to remove non-specifically bound oligonucleotide, the membrane was blocked in 1X Denhardt's blocking reagent for 30 min. The membrane was then incubated in 1X Denhardt's blocking reagent containing anti-digoxigenin-alkaline phosphatase for 1 h. The signal was detected with a digoxigenin luminescent detection kit (Roche), exposed to X-ray film, and scanned. The mean TRF length was determined as mean TRF length = ∑(ODi)/∑(ODi/Li),where ODi is the total signal intensity in interval i and Li is the molecular weight at the mid-point of interval i.

To correlate TRF length assay with real-time PCR-based telomere assay, the reference DNA and nine randomly-selected DNA samples were tested with these two techniques to determine the mean TRF length and the T/S ratio. The correlation was then examined by comparing the results of these two techniques with linear regression analysis.

### Data analysis

Data were presented as mean±SD or median (interquartile ranges) unless otherwise stated. For continuous data with abnormal distribution, including age of the puerperal, neonate body length, neonate body mass, Apgar score, gestational age, placental cadmium concentration and placental lead concentration in the control group or Guiyu group, Nonparametric Mann-Whitney U test was used to analyze the differences of the data between the two groups. For categorical data neonate's gender composition, Chi-square test was used to compare the difference of gender composition in the two groups. Due to the fact that data distributions of placental cadmium concentration and placental lead concentration were abnormal, Spearman correlation analysis was used to investigate the correlations of placental cadmium and lead concentrations with placental telomere length, with corrections for smoking history. For the same reason, Spearman correlation analysis was use to investigate the correlations of birth weight and Apgar score with placental cadmium concentration. To further analyze the correlation of placental cadmium (or lead) concentration with placental telomere length, quartile division was used. First, all the data pairs were sorted in ascending order by placental cadmium (or lead) concentration, divided into four quartiles with the 25th, the 50th and the 75th percentile of placental cadmium (or lead) concentration as the three cutting points. The correlation was then analyzed by investigating the variation trend of the mean placental telomere length of the four quartiles as the placental cadmium (or lead) concentration range of each quartile increased. To analyze the differences of the placental telomere length of the four quartiles, SNK test was used to conduct multiple comparisons under the circumstance of homoscedasticity. The quartile division was also used to investigate the correlations of birth weight and Apgar score with placental cadmium concentration. To better relate subjects of Guiyu group with high level of placental cadmium pollution, quartile division based on placental cadmium concentration was used to divide all the subjects of Guiyu group and the control group into four quartiles. Starting from the 1st quartile of the lowest placental cadmium concentration range to the 4th quartile of the highest placental cadmium concentration range, the number of subjects from Guiyu group and that from the control group were first determined in each of the four quartiles. Chi-square was then used to compare the difference of these two numbers (categorical data) in each of the four quartiles. Trend Chi-square test was also used to explore the variation trend of these two numbers along the four quartiles. To explore the influencing factors for placental telomere length, we chose the mean of placental telomere length of the control group as the cut point to define placental telomere length as a binomial variable to meet the requirement of unconditional logistic regression analysis, which was employed to analyze the data. Given the fact that placental telomere length is continuous distribution, not binomial distribution, defining placental telomere length as a binomial variable may result in loss of information. In order to better harness the useful information of placental telomere length, we used Spearman correlation analysis to investigate the correlation of placental cadmium and lead concentrations with placental telomere length. To investigate the correlation of the mean TRF length determined with TRF length assay and T/S ratio assessed with real-time PCR-based telomere assay, linear regression analysis was used. To assure that digestion of intact DNA of placenta did not lead to preferential loss of the telomere and a skewed data distribution of telomere length, One-Sample Kolmogorov-Smirnov Test was used to test the normality of data distribution of telomere length. A p<0.05 was considered statistically significant. All data analysis used SPSS software (SPSS Inc., Chicago, IL). The powers of all the tests in each subgroup were over 80% calculated with NCSS-PASS (NCSS Inc., USA, 2005 version).

## Results

### General characteristics of the study subjects

General characteristics of the study subjects are shown in Table 1. Guiyu group and the control group did not differ in age of the puerpera and neonates' gender composition. Whereas, significant differences were found in neonate body length, neonate body mass, Apgar score and gestational age between the two groups with newborns of the Guiyu group showing significantly less well development than the control group.

**Table 1:** General characteristics of the study subjects in Guiyu group and the control group.

Characteristics	Control group (n = 93)	Guiyu group (n = 227)
Age of the puerpera (Years)	27.63±4.649	26.45±4.309
Neonate body length (cm)	50.23±1.680	51.09±2.117^*^
Neonate body mass (kg)	3.3187±0.46553	3.1488±0.38974^*^
Apgar score	9.98±0.147	9.88±0.445^**^
Gestational age (weeks )	39.28±1.361	39.69±1.088^*^
Male	54.7%	51.1%
Female	45.3%	48.9%

Data were expressed as mean±SD or percentage. *: p<0.05, Guiyu group vs. the control group, **: p<0.01, Guiyu group vs. the control group.

### Correlation of placental cadmium and lead concentrations with placental telomere length

Spearman correlation analysis revealed that placental telomere length was negatively correlated with placental cadmium concentration (r = −0.138, p = 0.013, Table 2). This negative correlation became even more evident if the data was analyzed with quartile division described in the data analysis section. As indicated in Table 3, the mean of placental telomere length dwindled progressively as the placental cadmium concentration range in each quartile increased. It is noteworthy that the mean length of placental telomere of the 2^nd^, the 3^rd^ and the 4^th^ quartiles was all significantly shorter than that of the 1^st^ quartile (p<0.05), but no significant difference was found between the 2^nd^ and the 3^rd^, or between the 3^rd^ and the 4^th^ quartiles (p all>0.05, Table 3). However, no correlation between placental lead concentration and placental telomere length was detected (r = 0.027, p = 0.639, Table 2), which was also confirmed by the results of quartile division.

**Table 2:** Spearman correlation analysis of placental cadmium concentration and placental lead concentration with placental telomere length.

Related factors	Placental telomere length	
	r	p value
Placental cadmium concentration (n = 320)	−0.138	0.013*
Placental lead concentration (n = 309)	0.027	0.639

* : p<0.05.

**Table 3:** Correlation analysis of placental cadmium concentration and placental telomere length based on quartile division.

Order of quartile	N	Range of placental cadmium concentration	Mean of placental telomere length±SD
1^st^ quartile	80	0.000607∼ µg/g*	1.0560±0.28115*
2^nd^ quartile	80	0.0294∼ µg/g	0.9456±0.26776
3^rd^ quartile	80	0.0764∼ µg/g	0.9410±0.27924
4^th^ quartile	80	0.1261∼2.95 µg/g	0.9309±0.26075

* : p<0.05, the 1^st^ quartile vs. the 2^nd^, the 3^rd^ and the 4^th^ quartile. Multiple comparisons were done using SNK test.

### Placental cadmium and lead concentrations in Guiyu group and the control group

The medians of placental cadmium concentration were 0.0929 (P_25_–P_75_: 0.0640–0.1432) µg/g for Guiyu group and 0.0239 (P_25_–P_75_: 0.0167–0.0458) µg/g for the control group, showing significant difference between the two groups (p<0.01, Figure 1B). The medians of placental lead concentration was 1.2491 (P_25_–P_75_: 0.6681–2.5901) µg/g for Guiyu group and 1.3525 (P_25_–P_75_: 0.6823–4.1111) µg/g for the control group respectively. No significant difference was found between the two groups (Figure 1C).

In addition, as indicated in Table 4, the placental cadmium concentration ranges in the 2^nd^, the 3^rd^ and the 4^th^ quartiles were all significantly higher than that of the 1^st^ quartile (p<0.05). As shown in Table 4, 61 subjects from the control group segregated into the 1^st^ quartile, of which the placental cadmium concentration range was the lowest in this study. However, 18, 8 and 6 subjects from the control group segregated into the 2^nd^, the 3^rd^ and the 4^th^ quartile respectively, of which the placental cadmium concentration ranges were all significantly higher than that of the 1^st^ quartile (p<0.05). Hence, 84.9% of the subjects from the control group corresponded to lower placental cadmium concentration. On the contrary, only 19 subjects from Guiyu group clustered in the 1^st^ quartile, whereas, 62, 72 and 74 subjects from Guiyu group clustered in the 2^nd^, the 3^rd^ and the 4^th^ quartile respectively. Thus, in sharp contrast to the control group, vast majorities of the subjects from Guiyu group corresponded to higher placental cadmium concentration. This was confirmed by the result of Chi-square test shown in Table 4 (p _Pearson Chi-square_<0.001, p _trend_<0.001).

**Table 4:** Additional correlation analysis of cadmium pollution with Guiyu group and the control group.

Order of the quartile	Range of placental cadmium concentration	Total Number	Control group		Guiyu group		p _Pearson Chi-Square_	p _trend_
			N	percentage	N	percentage		
The 1^st^ quartile	0.000607∼ µg/g*	80	61	(76.2%)	19	(23.8%)		
The 2^nd^ quartile	0.0294∼ µg/g	80	18	(22.5%)	62	(77.5%)	0.001	0.001
The 3^rd^ quartile	0.0764∼ µg/g	80	8	(10.0%)	72	(90.0%)		
The 4^th^ quartile	0.1261∼2.95 µg/g	80	6	(7.5%)	74	(92.5%)		

* : p<0.05, the 1^st^ quartile vs. the 2^nd^, the 3^rd^ and the 4^th^ quartile.

### Influencing factors for placental telomere length

Ten factors, including mother's age, Guiyu as residence during pregnancy, whether or not the pregnant woman had a cold during pregnancy, mother's involvement in e-waste recycling, father's involvement in e-waste recycling, mother's smoking status, mother's passive smoking status, mother's drinking status, gestational age and neonates' gender, were selected and unconditional logistic regression analysis was performed to screen the influencing factors. As shown in Table 5, the factor “Guiyu as residence during mother's pregnancy” was statistically associated with placental telomere length (OR = 0.200, 95% CI: 0.067–0.599, p<0.01). In addition, it should be noted that another factor “mother's health status during pregnancy” reached p value of 0.061, near but not quite the level of statistical significance.

**Table 5:** Exploration of influencing factors for placental telomere length based on unconditional logistic regression analysis.

Investigated factors	Long or short placental telomere length^a^ (n = 171)			
	B	Wald	p value	OR (95% CI)
Guiyu as residence during pregnancy	−1.608	8.267	0.004^*^	0.200 (0.067, 0.599)
Mother's health status during pregnancy	−0.957	3.521	0.061	0.384 (0.141, 1.043)

a: long or short placental telomere length, taking the mean of placental telomere length of the control group as the cutting point, *: p<0.01.

### Correlation of the mean TRF length and T/S ratio

**Figure 2:**
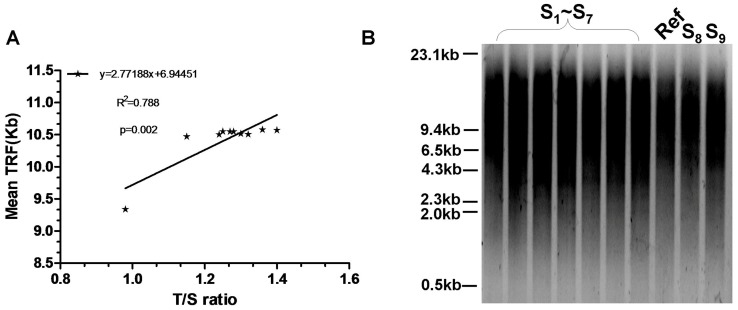
Correlation of T/S ratio and the mean terminal restriction fragment (TRF) length. (A) Correlation of T/S ratio assessed by real-time PCR-based telomere assay and the mean TRF length determined by southern blotting in ten DNA samples. The linear regression line that best fit the data (p = 0.002) is shown. (B) The mean terminal restriction fragment (TRF) length was assessed by southern blotting. Size of molecular weight markers (Kb) is shown at the left side. S_1_–S_9_: nine randomly-selected DNA samples, Ref: the reference DNA used in real-time PCR-based telomere assay.

As shown in Figure 2, the mean TRF length determined with southern blotting was strongly correlated with T/S ration assessed with real-time PCR-based telomere assay. Linear regression analysis revealed that the correlation coefficient R^2^ for T/S ration and the mean TRF length was 0.788, and the p value was 0.002. The telomere repeat copy number for the reference DNA was approximately 9.34 Kb.

### Correlation of birth weight and Apgar score with placental cadmium concentration based on quartile division

Birth weight was found to correlate with quartile of cadmium concentration as confirmed by Spearmen correlation analysis (p = 0.017). Apgar score was not found to correlate with quartile of cadmium concentration.

### Effect of DNA digestion on placental telomere length

To assure that digestion of intact DNA of placenta did not lead to preferential loss of the telomere and a skewed data distribution of telomere length, we analyzed the data distribution of placental telomere length with One-Sample Kolmogorov-Smirnov Test. One-Sample Kolmogorov-Smirnov Test demonstrated that the data distribution of placental telomere length was normally distributed (p = 0.203). This result indicated that digestion of intact DNA of placenta did not lead to preferential loss of the telomere and a skewed data distribution.

## Discussion

Our results demonstrated that placental telomere length was negatively correlated with placental cadmium concentration with a Spearman correlation coefficient of −0.138 and a p value of 0.013. The negative correlation was more evident if the data were analyzed with quartile division described in the data analysis section. The negative correlation is corroborated by a recent in vitro study with mouse that reported embryo exposed to cadmium chloride at 5–100 µM (i.e. 0.92–18.3 µg/ml) exhibited increased oxidative stress, chromosomal instability and telomere attrition and loss [29]. Cadmium chloride at 0.92–18.3 µg/ml of the above study equals to 0.92–18.3 µg/g because it was dissolved in water [29]. Cadmium chloride at 0.92–18.3 µg/g is in correspondence with the placental cadmium concentration range of the 4^th^ quartile of our study (Table 3).

The negative correlation we observed becomes more meaningful biologically when taking into considerations of the possible mutagenic effects of cadmium on telomere. Cadmium is a well-established mutagen and several mechanisms for its mutagenicity have been proposed, including modulation of gene expression [30], signal transduction [31], and inhibition of methylation [32]. Two mechanisms are predominately important, i.e., induction of reactive oxygen species and inhibition of DNA repair [33]–[35]. Cadmium, on the one hand, through inhibition of cellular antioxidant enzymes [36], [37], can cause abundant accumulations of reactive oxygen species in cell and eventually give rise to DNA damages. And on the other, by replacing zinc ions in DNA repair enzymes, Cadmium can inactivate DNA repair enzymes and deprive of the capabilities of cells to repair DNA damages caused by reactive oxygen species (ROS) [38], [39]. Telomeric DNA is more vulnerable to ROS attack than other genomic elements because of its special compositions (a triple-G-containing structure) [40], [41], and is irreparable according to a recent study by Fumagalli et al [42]. Oxidative stress accelerates telomere attrition [43], whereas antioxidant slows down this process [44], [45]. Combining these findings, it would not be difficult to comprehend the negative correlation observed in our study.

Moreover, as indicated in Table 3, the range of the placental cadmium concentration of the 1^st^ quartile was between 0.000607 and 0.0249 ug/g which was in good accordance with the normal background tissue level previously reported [11], [46], suggesting that subjects of the 1^st^ quartile were good representatives of the unexposed population. Thus, the mean of placental telomere length in the 1^st^ quartile more or less reflected the normal placental telomere length of the unexposed population. Starting from the 2^nd^ quartile, the mean of placental telomere length descended at a high rate first and at a low rate subsequently. Given that the mean of placental telomere length in the 1^st^ quartile was a representation of the unexposed population, it is likely that the cutting point of cadmium concentration between the 1^st^ and the 2^nd^ quartile (0.0294 µg/g) might be around the tipping point at which attrition of the placental telomere commenced. This critical concentration of tissue cadmium at which attrition of the human telomere commenced has not been reported previously. This critical concentration might serve as a reference to evaluate human cadmium pollution.

However, differing from the negative correlation found between placental cadmium concentration and placental telomere length, no correlation was found between placental telomere length and placental lead concentration in this study, which was also confirmed by the result of quartile division. An in vitro study by Liu et al reported that 0.5 µM lead acetate caused telomere attrition in cultured human hepatocyte cell line L-02 [47]. The difference between their finding in cultured cells and ours in tissue might be caused by the concentration and duration of exposure of the cells or tissue to lead. Placenta is a more effective barrier to cadmium than to mercury and lead, leading to accumulation of cadmium but not lead in the placental tissue [48]. Cadmium level in the umbilical cord blood was reported to be about 70% of that in the maternal blood, but the lead level in the umbilical cord blood was the same as that in the maternal blood [49], [50]. Hence, lead accumulates to a lesser extent than cadmium does in the placenta. This may provide an explanation to the difference between cadmium and lead pollution in their effects to placental telomere length.

It should be noted that other pollutants of e-waste were also found in the placentas from Guiyu residents, e.g., chromium, nickel, PCBs, PBDEs and PAHs [2], [11], [51], [52]. Additionally, PAHs have been shown to be capable of causing telomere attrition in peripheral blood lymphocytes of cokeoven workers [53]. It would be desirable if the strength of correlation found in this study could be increased with a larger sample size and cadmium could be compared to other pollutants in the placenta. However, we had exhausted our collected placental samples. Collection of placental samples and cord blood in Guiyu has been a challenging task due to the impediments from the local residents and officials who regard the exposure of e-waste pollutions as a negative taboo to their community. In addition, the situations of e-waste pollutions in Guiyu have been improving over the years and the level of pollution has changed. It would have been incomparable if additional samples were collected to include in this study. Prior to this study, the same set of placental samples had been used to carry out several other research projects. The remaining precious placental samples were only sufficient for us to examine two pollutants (i. e. cadmium and lead). More importantly, the decision to assess cadmium and lead in the placentas was based on our previous study which showed that cadmium and lead were the predominant toxic heavy metals in the placentas in Guiyu [11]. Our study cannot completely rule out the possibility that other pollutants of e-waste, along with cadmium, might also have contributed to the short telomere observed. Our findings support the notion that cadmium is associated with short telomere. This is the first report to demonstrate a direct link between tissue cadmium pollution and tissue telomere length in humans.

As the telomere was extracted from the placenta, which is a derivative of fetal embryo, it is likely that the fetus and the newborn would be similarly affected. One should be alarmed to the consequences of high tissue cadmium concentration in liveborn offsprings in e-waste polluted area. There has been a surge in cases of child leukemia in Guiyu, which could be partly caused by shortened telomere of the fetus and newborn, as corroborated by several reports [54]–[57]. Adamson et al found significantly shortened telomere and oncogene activation in acute leukemia in children [56]. Hartmann et al also suggested that telomere attrition played an important role in the development of genetic instability during the pathogenesis of acute myeloid leukemia [57]. Telomere attrition has been suggested to be an accelerator for the onset of aging-related pathology [26]. Therefore, offspring in e-waste polluted area might experience aging-related pathology earlier and have shortened life span. In addition, telomere length has been known to be paternally inheritable, implying that in population of e-waste polluted area shortened telomere could be passed down the generations with lasting negative consequences [58].

## Conclusions

We showed that placental cadmium concentration was negatively correlated with placental telomere length. Placental cadmium concentration of 0.0294 µg/g might be the in vivo tipping point at which attrition of placental telomere commenced. No correlation between placental lead concentration and placental telomere length was detected in this study. Therefore, our data demonstrate that exposure to cadmium pollution during pregnancy may be a risk factor of shortened placental telomere length with related lasting medical and social consequences.

## Supporting Information

Table S1 **Primers used for assessment of placental telomere length.**
(DOC)
