# Supplementary material for: Short Placental Telomere was Associated with Cadmium Pollution in an Electronic Waste Recycling Town in China
Source: PLoS One. 2013 Apr 2;8(4):e60815. doi: 10.1371/journal.pone.0060815 (PMC3614985; doi:10.1371/journal.pone.0060815)
Supplement: Table S1 — Primers used for assessment of placental telomere length. (DOC) [file pone.0060815.s001.doc]

**Table S1.** Primers used for assessment of placental telomere length.

| **Primer name** | **Primer sequence 5’-3’** | **Thermal cycling profile** |
| --- | --- | --- |
| Telomere | (Tel 1) GGTTTTTGAGGGTGAGGGTGAGGGTGAGGGTGAGGGT | 95℃ 30sec, 30 cycles of 95℃ 5sec, 54℃ 2min |
|  | (Tel 2) TCCCGACTATCCCTATCCCTATCCCTATCCCTATCCCTA |  |
| 36B4 | (36B4d) CCCATTCTATCATCAACGGGTACAA | 95℃ 30sec, 40 cycles of 95℃ 5sec, 58℃ 1min |
|  | (36B4u) CAGCAAGTGGGAAGGTGTAATCC |  |
